# Potentiation of Amitriptyline Anti-Hyperalgesic-Like Action By Astroglial Connexin 43 Inhibition in Neuropathic Rats

**DOI:** 10.1038/srep38766

**Published:** 2016-12-12

**Authors:** Tiffany Jeanson, Adeline Duchêne, Damien Richard, Sylvie Bourgoin, Christèle Picoli, Pascal Ezan, Franck Mouthon, Christian Giaume, Michel Hamon, Mathieu Charvériat

**Affiliations:** 1 Theranexus, Lyon, France; 2 CIRB, Collège de France, Paris, France; 3 CHU Clermont-Ferrand, Service de Pharmacologie Médicale, Clermont-Ferrand, France; 4 INSERM U894 – CPN, Paris, France

## Abstract

*Antidepressants, prescribed as first line treatment of neuropathic pain, have a limited efficacy and poorly tolerated side effects. Because recent studies pointed out the implication of astroglial connexins (Cx) in both neuropathic pain and antidepressive treatment, we investigated whether their blockade by mefloquine could modulate the action of the tricyclic antidepressant amitriptyline. Using primary cultures, we found that both mefloquine and amitriptyline inhibited Cx43-containing gap junctions, and that the drug combination acted synergically. We then investigated whether mefloquine could enhance amitriptyline efficacy in a preclinical model of neuropathic pain. Sprague-Dawley rats that underwent chronic unilateral constriction injury (CCI) to the sciatic nerve (SN) were treated with either amitriptyline, mefloquine or the combination of both drugs. Whereas acute treatments were ineffective, chronic administration of amitriptyline reduced CCI-SN-induced hyperalgesia-like behavior, and this effect was markedly enhanced by co-administration of mefloquine, which was inactive on its own. No pharmacokinetic interactions between both drugs were observed and CCI-SN-induced neuroinflammatory and glial activation markers remained unaffected by these treatments in dorsal root ganglia and spinal cord.* Mechanisms downstream of CCI-SN-induced neuroinflammation and glial activation might therefore be targeted. Connexin inhibition in astroglia could represent a promising approach towards improving neuropathic pain therapy by antidepressants.

Neuropathic pain, caused by lesion or dysfunction of the peripheral or central nervous system^1^, substantially affects the quality of life and is associated with heavy individual and societal burden^2^. Available treatments with anticonvulsants, antidepressants, opioids, and lidocaine or capsaicin patches are only moderately effective and may induce poorly tolerated side effects that negatively impact compliance^1^,^2^,^3^,^4^,^5^. Numerous peripheral and central pathways have been suggested as new therapeutic avenues^6^; this study focused on a recently proposed target, astroglial connexins (Cxs), since these “gap junction proteins” have been reported to be involved in neuropathic pain^7^ and as a target for antidepressants^8^.

Connexins are transmembrane proteins, assembled as hexamers, called hemichannels (HCs) that, when open, allow direct communication between the cytoplasm and the extracellular space^9^,^10^,^11^,^12^. Hemichannels of adjacent cells can join to form gap junctions channels (GJCs), through which direct cytoplasm-to-cytoplasm exchanges occur^9^,^10^,^11^. A large variety of ions and signaling molecules (<1 kDa) can diffuse through GJCs, which play therefore a key role in extensive ionic and biochemical exchanges between cells^9^,^10^,^11^. In the brain, astrocytes’ coupling through GJC channels contributes to ionic homeostasis maintenance and to various biochemical/metabolic processes^9^. On the other hand, HCs are involved in astrocyte release of gliotransmitters^12^,^13^, uptake of glucose^14^ and efflux of glutathione^15^. In astrocytes, two major Cxs have been identified, Cx43 and Cx30, both contributing to GJC. However, so far only Cx43-containing HCs have been shown to operate in astrocytes^16^.

Under peripheral and central neuropathic conditions, a marked increase in Cx43 levels was reported in both dorsal root ganglia (DRG) and spinal cord tissues, during pain induction phase and its maintenance^7^,^17^. Concomitant functional increases of Cx43-containing GJCs^18^,^19^ and HCs^20^ promote astrocyte coupling and enhance HC-mediated release of excitatory gliotransmitters, including glutamate^21^,^22^ and ATP^23^, that activate post-synaptic NMDA receptors and purinergic receptors, respectively. Contribution of this signaling sequence to pain sensitization mechanisms is supported by data showing that inactivation of Cx43-mediated functions by pharmacological^20^,^24^,^25^ or genetic approaches^26^ reduces hyperalgesia-and allodynia-like behaviours in validated models of neuropathic pain. Furthermore, antidepressants used to alleviate neuropathic pain, such as duloxetine^27^ and amitriptyline^28^, were reported to affect Cx43 expression and function in astrocytes^8^.

We first investigated the effects of the widely used antidepressant, amitriptyline, on Cx43 expression and functions in astrocytes. Then, using the validated model of neuropathic pain that consists of unilateral ligation of the sciatic nerve in rats^29^, we explored whether partial Cx43 channels blockade by mefloquine, a potent connexin blocker^30^,^31^, could interfere with the anti-hyperalgesic-like action of amitriptyline. Finally, whether pharmacokinetic and/or pharmacodynamic interactions accounted for the modulatory effect of mefloquine on amitriptyline-induced effect was investigated using relevant HPLC and real time RT-qPCR quantifications.

## Results

### Mefloquine and amitriptyline inhibited Cx43-mediated channel functions in rat cortex astrocytes

**Figure 1:**
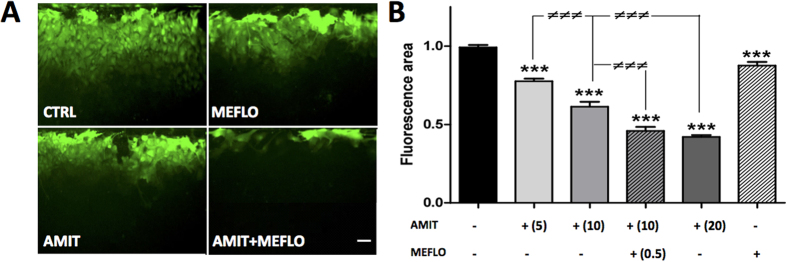
Synergic inhibitory effects of mefloquine and amitriptyline on astroglial Cx43 coupling. Cultured astrocytes were treated during 24 h before imaging. (**A**) Typical fluorescence photomicrographs of intercellular Lucifer yellow spreading after a 10-min scrape loading under the following conditions: no treatment (control, CTRL, black bar); mefloquine (MEFLO 0.5 μM); amitriptyline (AMIT 10 μM); AMIT 10 μM + MEFLO 0.5 μM. Calibration scale: 20 μm. (**B**) Fluorescence area of Lucifer yellow spreading after 24 h treatment with AMIT and/or MEFLO at the indicated concentrations (μM). Values are expressed with respect to CTRL fluorescence area. Each bar is the mean ± S.E.M. of 3–6 independent determinations. ***P < 0.001 compared with CTRL group, one-way ANOVA, Newman-Keuls; ^≠≠≠^*P* < 0.001 comparison between AMIT-treated groups, one-way ANOVA, Newman-Keuls test.

Primary cultures of astrocytes only express Cx43^32^,^33^. After a 24 h exposure to mefloquine (MEFLO) at 0.5 μM, Cx43-mediated astrocyte coupling assessed from LY fluorescence spreading was significantly reduced (Fig. 1A,B; *P* < 0.001, One-way ANOVA, Newman-Keuls test), as expected from GJC inhibition by this drug^30^,^31^. Under the same conditions, amitriptyline (AMIT) was also found to inhibit Cx43-mediated astrocyte coupling in a concentration-dependent manner: by 22% (n = 4) at 5 μM, 27% (n = 5) at 10 μM, 58% (n = 4) at 20 μM (Fig. 1B). Interestingly, the relative decrease in LY spreading fluorescence evoked by AMIT at 10 μM reached −52% in the presence of MEFLO (Fig. 1B), a percentage almost twice as high as that found for the tricyclic drug tested alone at this same concentration (see above).

**Figure 2:**
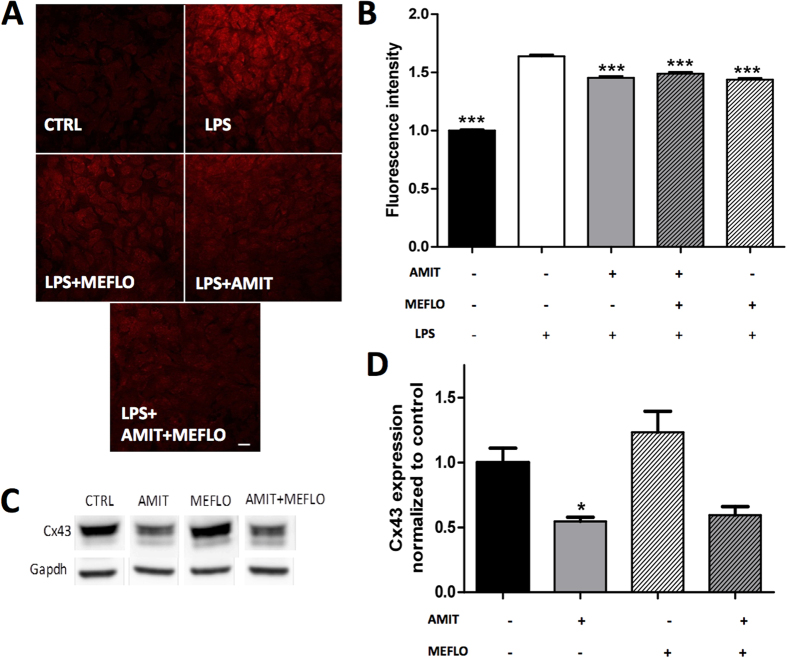
Unchanged inhibitory effect of amitriptyline on astroglial Cx43 expression and hemichannels upon concomitant treatment with mefloquine. (**A**) Photomicrographs of fixed cells examined at 40× with a confocal laser-scanning microscope one day after EtBr uptake in cultured astrocytes treated with: none (control, CTRL, black bar); LPS (1 μg/mL) alone; LPS + MEFLO 0.5 μM; LPS + AMIT 10 μM; LPS + AMIT 10 μM + MEFLO 0.5 μM. Stacks of 10 consecutive confocal images were taken at 0.49 μm intervals. Calibration scale: 20 μm. (**B**) Effects of AMIT and/or MEFLO on LPS-induced HeC activity measured by EtBr uptake (fluorescence intensity). Values are expressed with respect to CTRL fluorescence intensity. Each bar is the mean ± S.E.M. of 4–6 independent determinations. ***P < 0.001 compared with treatment with LPS alone, Kruskal-Wallis, Dunn’s test. (**C**) Western blots of Cx43 and Gapdh in extracts from cultured astrocytes after 24-hour treatment by amitriptyline 10 μM and/or mefloquine 0.5 μM. (**D**) Quantification of Cx43 immunolabeling under these respective treatment conditions. Each bar is the mean ± S.E.M. of 5 independent determinations. *P < 0.05, as compared with control (no treatment, black bar) (Kruskal-Wallis, Dunn’s test).

For assessment of the effects of MEFLO and/or AMIT on HC-mediated EtBr uptake, cortical astrocytes were first treated with LPS to enhance HC activity^14^. As expected, LPS significantly increased EtBr uptake by 64% (n = 6) compared to basal situation (Fig. 2B), up to levels high enough for reliable quantification of possible modulations by drugs. Data depicted in Fig. 2 show that a 24 h exposure to MEFLO (0.5 μM) or AMIT (10 μM) significantly reduced EtBr uptake in astrocytes which had been pretreated with LPS, indicating that both drugs inhibited LPS-induced HC activity (n = 6 per condition, *P* < 0.001 as compared with LPS treatment alone, one-way ANOVA, Dunn test). However, in contrast to that observed on GJC (Fig. 1B), no synergic interaction between these drugs was noted on HC activity because the reduction in EtBr uptake induced by the combination of MEFLO + AMIT did not differ from that evoked by either drug tested alone (Fig. 2B).

### Treatment with amitriptyline but not mefloquine decreased Cx43 expression in rat cortex astrocytes

Under the same 24 h treatment conditions as those producing reduced GJ and HC functions, AMIT (10 μM) but not MEFLO (0.5 μM) significantly reduced the levels of Cx43 protein measured by western blots in extracts from treated cortex astrocyte cultures. As shown in Fig. 2D, AMIT-induced effect reached −45% compared to CTRL conditions (*P *< 0.05, Kruskal-Wallis test, Dunn’s post test). The combination of AMIT + MEFLO also induced a decrease of Cx43 expression (−40% vs CTRL), but statistical significance was not reached compared to CTRL conditions (*P *> 0.05, Kruskal-Wallis test, Dunn’s post test).

### Acute administration of mefloquine and/or amitriptyline did not affect mechanical hyperalgesia-like behaviour in neuropathic CCI-SN rats

**Figure 3:**
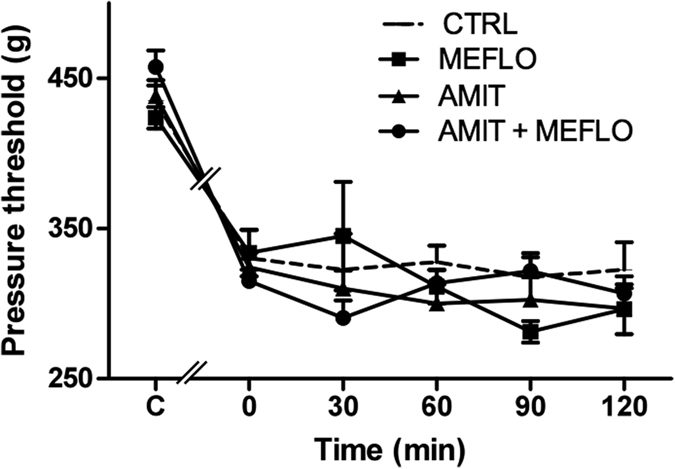
Unaltered CCI-SN-induced mechanical hyperalgesia after acute treatment with amitriptyline and/or mefloquine. Amitriptyline (AMIT, 12 mg/kg i.p.) or its vehicle (saline) was co-administered with mefloquine (MEFLO, 1 mg/kg i.p.) or its vehicle (saline) in rats that had undergone unilateral CCI-SN two weeks before. Mechanical hyperalgesia was assessed at various times after treatment (abscissa) by determining pressure threshold value (in g) to trigger vocalization in the Randall-Selitto test. Each point is the mean ± S.E.M. of independent determinations in n rats: CTRL (saline + saline), n = 6; AMIT, n = 9; MEFLO, n = 4; AMIT + MEFLO, n = 10). C on abscissa: control naïve rats before CCI-SN; 0 on abscissa: two weeks after CCI-SN just prior to treatments. None of the pressure threshold values determined after treatment significantly differed from those at time 0; two-way ANOVA, Bonferroni test.

A marked decrease in pressure threshold values to trigger hind paw withdrawal (not shown) and vocalization (Fig. 3, *P *< 0.05, two-way ANOVA, Bonferroni post test) in the Randall-Selitto test was noted in 75% (n = 29) of operated rats two weeks after unilateral ligation of the sciatic nerve, as expected of the occurrence of CCI-SN-induced hypersensitivity to mechanical stimulation^34^,^35^. Performance of the Randall-Selitto test at various times (up to 2 hours) after acute administration of MEFLO (1 mg/kg i.p.), AMIT (12 mg/kg i.p.) or the combination of both drugs did not reveal any significant changes in these pressure threshold values (Fig. 3).

### Anti-hyperalgesic-like effects of chronic amitriptyline treatment were enhanced by combined chronic administration of mefloquine in neuropathic CCI-SN rats

**Figure 4:**
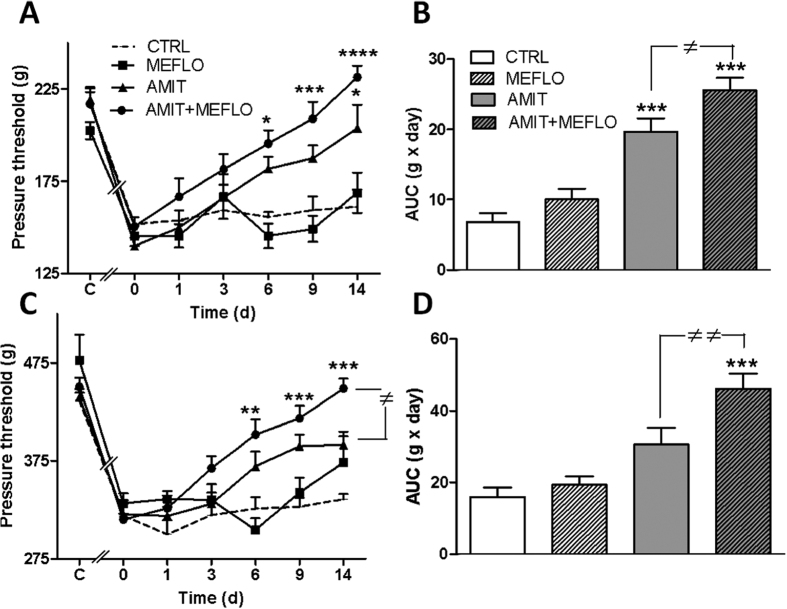
Potentiation by mefloquine of the anti-hyperalgesic effect of chronic treatment with amitriptyline in CCI-SN rats. Rats underwent unilateral CCI-SN and two-week-treatments with amitriptyline (12 mg/kg s.c. daily, via osmotic mini-pump) or its vehicle (saline s.c. via osmotic mini-pump), and mefloquine (0.5 mg/kg i.p., twice daily) or its vehicle (saline) started on day 15 post-surgery. Mechanical hyperalgesia was assessed by determining pressure threshold values to trigger hindpaw withdrawal (**A**,**B**) and vocalization (**C**,**D**) in the Randall-Selitto test performed at various times during treatment (abscissa, in days). (**A**,**C**) Time-course changes in pressure threshold values. Each point is the mean ± S.E.M. of independent determinations in n rats (“AMIT + MEFLO”, n = 14; “AMIT”, n = 12; “MEFLO”, n = 10; “CTRL”, n = 8). C on abscissa: control rats before CCI-SN; 0 on abscissa: two weeks after CCI-SN just prior to treatments. (**B**,**D**) AUC values (g × day) calculated from respective time course-curves illustrated in A, C. Each bar is the mean ± S.E.M. of n rats. *P < 0.05, **P < 0.01, ***P < 0.001 compared with CTRL group, two-way ANOVA, Bonferroni test; ^≠^*P* < 0.05, ^≠≠^*P* < 0.01, one-way ANOVA, Newman-Keuls test.

In contrast to its lack of effect under acute treatment conditions, continuous administration of AMIT at the dose of 12 mg/kg/day (through a s.c. implanted Alzet minipump) for 14 days produced a progressive, time-dependent, increase of pressure threshold values to trigger hindpaw withdrawal (Fig. 4A) and vocalization (Fig. 4C) in CCI-SN rats. At the end of the 14 day-treatment, pressure threshold values reached levels corresponding to 50–75% recovery in comparison with control values determined before CCI-SN surgery (C on abscissa; n = 8–14 in each group, *P *< 0.05, two-way ANOVA, Bonferroni test). In contrast, repeated daily administration of MEFLO (1 mg/kg i.p./day) was essentially inactive for the two-week treatment, as only a (non significant) tendency to elevated pressure threshold to trigger vocalization was noted on day 14 (Fig. 4C). However, when combined with AMIT, MEFLO, at the same inactive dose, significantly enhanced the anti-hyperalgesic-like effect of the tricyclic drug, as shown by the faster (Fig. 4A,C, *P *< 0.05 at days 6, 9, 14, two-way ANOVA, Bonferroni test) and larger (Fig. 4B,D, *P *< 0.05, one-way ANOVA, Newman-Keuls test) recovery toward normal pressure threshold values (C on abscissa) in CCI-SN rats treated with AMIT + MEFLO compared to AMIT alone (Fig. 4A,B,C,D). Indeed, at the end of the two week-treatment, pressure threshold values to trigger hindpaw withdrawal and vocalization no longer differed in [AMIT + MEFLO]-treated- vs control-CCI-SN rats (Fig. 4A,C).

### Mefloquine co-treatment did not affect brain and serum levels of amitriptyline after a 14 day-treatment

**Figure 5:**
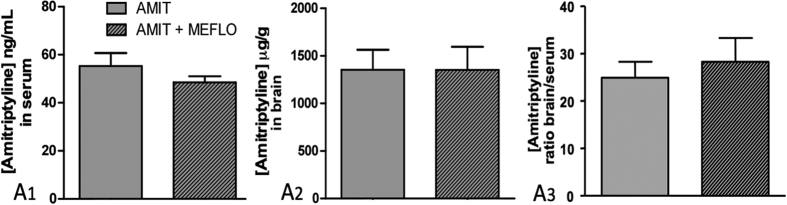
Unchanged serum and brain levels of amitriptyline by co-administration of mefloquine in CCI-SN rats after a two-week treatment with amitriptyline. Amitriptyline (12 mg/kg s.c. daily, via osmotic mini-pump) was co-administered with mefloquine (0.5 mg/kg i.p., twice daily, AMIT + MEFLO) or its vehicle (0.9% NaCl, AMIT) for 14 days in rats which had undergone CCI-SN two weeks before. At the end of treatment, amitriptyline levels were measured in serum (ng/ml, A1) and in brain (μg/g, A2). The ratio of amitriptyline levels in serum over those in brain (A_3_) was also calculated for each rat. Each bar is the mean ± S.E.M. of independent determinations in 8 rats. No significant difference was noted between both treatment groups (unpaired t-test).

A marked accumulation of AMIT in brain tissues was observed at the end of the 14 day continuous treatment with this drug at the daily dose of 12 mg/kg s.c. delivered through an Alzet osomotic mini-pump since brain AMIT levels reached 1350 ± 210 μg/g (corresponding to 4.87 ± 0.76 μM; mean ± S.E.M., n = 8), a value about 25 fold higher than that in serum: 55.3 ± 5.4 ng/ml (corresponding to 199.35 ± 19.47 nM; mean ± S.E.M., n = 8; Fig. 5). Co-treatment with MEFLO (2 × 0.5 mg/kg i.p. daily) for the same period did not significantly (*P *> 0.05, Student’s t-test) affect AMIT levels in brain and serum (1350 ± 240 μg/g and 48.4 ± 2.6 ng/ml, respectively, means ± S.E.M., n = 8).

### Chronic treatments with amitriptyline and/or mefloquine did not affect CCI-SN-induced up regulation of glial and neuro-inflammation markers in dorsal root ganglia and spinal cord

**Figure 6:**
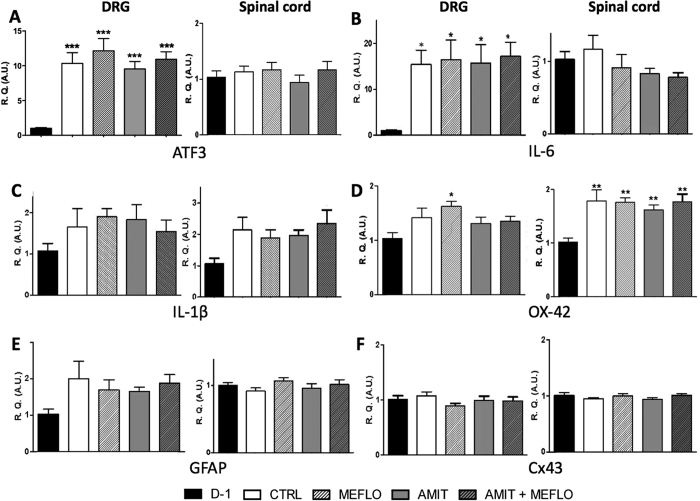
Unchanged CCI-SN-induced expression of mRNAs encoding glial and neuro-inflammatory markers in dorsal root ganglia and spinal cord in rats treated with amitriptyline and/or mefloquine or their vehicles for two weeks. Amitriptyline (AMIT, 12 mg/kg s.c. daily, via osmotic mini-pump) or its vehicle (CTRL, 0.9% NaCl) was co-administered with mefloquine (MEFLO, 0.5 mg/kg i.p., twice daily) or its vehicle (0.9% NaCl) for 14 days in rats which had undergone unilateral CCI-SN two weeks before. At the end of treatments, mRNA levels of ATF3 (**A**) IL-6 (**B**) IL-1β (**C**) OX-42 (**D**) GFAP (**E**) and Cx43 (**F**) encoding mRNAs were measured by RT-qPCR in ipsilateral dorsal root ganglia (L4-L6) and dorsal quadrant of the L4-L6 segment of the spinal cord. Determinations were also made in naïve rats (D-1, black bars) for comparison. Each bar is the mean ± S.E.M. of independent determinations in 5–10 rats. *P < 0.05, **P < 0.01, ***P < 0.001 compared with naïve rats (D-1, black bars), Kruskal-Wallis and one-way ANOVA, followed respectively by Dunn and Newman-Keuls tests. None of the treatments significantly changed CCI-SN-induced up regulation of any of those markers in both dorsal root ganglia and lumbar cord (comparison with CTRL CCI-SN rats, empty bars).

At the end of vehicle administrations for 14 days starting two weeks after surgery, i.e. 28 days after CCI-SN, marked increases in the levels of transcripts encoding ATF3 (Fig. 6A) and IL-6 (Fig. 6B) were noted in right (ipsilateral) L4-L6 DRG compared to those found in unoperated naive rats. A tendency to an up-regulation of DRG levels of mRNAs encoding IL-1β (Fig. 6C), OX-42 (Fig. 6D) and GFAP (Fig. 6E) was also observed in CCI-SN rats, whereas the expression of Cx43 (Fig. 6F), TNFα, BDNF and NR2B (not shown) did not differ from that quantified in unoperated animals.

CCI-SN-induced changes in L4-L6 spinal cord tissues were less pronounced than in DRG since only OX-42 (Fig. 6D) and IL-1β (Fig. 6C) transcripts were up regulated in operated vs naive unoperated animals.

As illustrated in Fig. 6, neither AMIT (12 mg/kg s.c. daily) or MEFLO (2 × 0.5 mg/kg i.p. daily) alone nor the combined administration of both drugs significantly modified CCI-SN-induced changes in the levels of transcripts encoding these various glial and neuroinflammation markers in DRG and spinal cord tissues.

## Discussion

Convergent data showed that Cx43 overexpression in DRG and spinal cord tissues contributes to the induction and maintenance of neuropathic pain, by promoting astrocyte coupling and enhancing the release of excitatory gliotransmitters by hemichannel opening at critical synaptic relays of pain signaling pathways^7^,^17^. Accordingly, Cx43 might be a potential target for alternative treatments of neuropathic pain. Indeed, high doses of Cx inhibitors have already been shown to reduce hyperalgesia- and allodynia-like behaviours in validated rodent models of neuropathic pain^36^. However, severe adverse effects are induced with such high dosages, which preclude the use of Cx inhibitors alone for developing treatments specifically targeted on neuropathic pain^27^,^37^,^38^. This led us to assess whether moderate pharmacological modulation of Cx43 functions in astrocyte GJCs and HCs by a relatively low dose of the Cx inhibitor mefloquine could enhance the anti-hyperalgesic efficacy of amitriptyline, a tricyclic antidepressant used as first line treatment of neuropathic pain^1^,^2^,^3^,^4^,^5^. To this goal, we performed both *in vitro* and *in vivo* studies, with primary cultures of rat cortical astrocytes on one hand and the rat model of neuropathic pain generated by unilateral ligation of the sciatic nerve on the other hand. Care was taken to use an amitriptyline dose (12 mg/kg/day) yielding brain and serum levels in the same μM range as that reached in humans treated with this drug^39^. On the other hand, the selected dose of mefloquine (1 mg/kg/day) led to a brain concentration (0.5 μM) high enough to affect GJ but largely lower than those producing therapeutic or adverse effects in humans^40^.

For *in vitro* experiments, astrocytes, with their typical pavement-like morphology under culture conditions^11^,^28^ (Figs 1 and 2), which markedly differs from the usual star or sponge-shape in brain tissues, were prepared from the cerebral cortex rather than the spinal cord in order to yield enough cell material. Indeed, previous studies showed that astroglial Cx43 GJCs behave similarly whether cultured astrocytes had been prepared from the cerebral cortex^14^,^41^ or the spinal cord^42^,^43^. This led us to infer that data obtained with cortical astrocytes would have relevance for spinal cord astrocytes, whose implication in neuropathic-like pain mechanisms has been extensively characterized in CCI-SN rats^34^,^44^. In addition to mefloquine, amitriptyline itself was found to decrease, in a concentration-dependent manner, Cx43-GJC-mediated Lucifer yellow spreading among cultured astrocytes, indicating clear-cut GJC inhibition by the tricyclic antidepressant. Interestingly, this effect, also related to down regulation of Cx43 expression, occurred at therapeutic μM concentrations reached in humans^45^. Recently, a similar inhibitory effect of GJCs by amitriptyline was reported in mouse cultured astrocytes^8^. In contrast, Morioka *et al*.^28^ reported that amitriptyline, at a slightly higher concentration, 25 μM and longer treatment time (48 h) up-regulated both Cx43 expression (mRNA and protein) and Cx43-mediated GJC function in cultured rat cortical astrocytes. Such discrepancy between these results and our own might be explained by differences in respective experimental conditions, notably for astrocyte cell cultures. In particular, the presence of microglial cells in astrocyte culture might be critical because previous studies showed that microglia strongly interacts with astrocytes to deeply modulate Cx activities^13^,^14^. Quantification of specific markers indicated that, under our culture conditions, microglial cells accounted for approximately 4% and 11% of all cells in rat and mouse models, respectively (not shown). However, further comparison with the discrepant data reported by Morioka *et al*.^28^ could not be made because the percentage of microglial cells was not determined in their study.

Interestingly, partial inhibition of GJC function by a low concentration of mefloquine (0.5 μM) was found to markedly enhance GJC inhibition by amitriptyline, whereas only weak, non additive, inhibitory effects of these drugs were noted on LPS-induced HC activity measured by astrocyte uptake of EtBr. This observation, suggesting the occurrence of some synergy between the *in vitro* effects of mefloquine and amitriptyline, led us to investigate whether a similar interaction might also occur *in vivo*. Indeed, mefloquine appeared especially relevant for such studies because it crosses the blood brain barrier^46^. Furthermore, its pharmacological profile indicates a relatively good selectivity as a Cx channel inhibitor. In particular, its affinity/potency at various serotonin (5-HT) and noradrenaline (NA) receptors and transporters mediating the anti-hyperalgesic and anti-allodynic effects of tricyclic antidepressants^47^ was low enough (EC_50_ = 9.3 μM on 5-HT_3_ receptors^48^; K_i_ > 9 μM and 3.8 μM on 5-HT_1A_ receptors and 5-HT_2C_ receptors, respectively^49^; EC_50_ > 1.9 μM on 5-HT_2A_ receptors ; EC_50_ > 5 μM on NA re-uptake^49^) to rule out any direct action of *in vivo* treatment at the dose of 1 mg/kg, leading to maximal mefloquine brain concentration around 0.5 μM^40^.

For investigating the effects of the combination of amitriptyline + mefloquine on neuropathic-like pain, we used the CCI-SN model of that consists of unilateral ligation of the sciatic nerve so as to reduce the nerve diameter and retard the epineurial circulation as described by Bennett and Xie^29^. Although Maves *et al*.^50^ previously emphasized that silk thread, in contrast to chromic gut thread, could not be used to produce mechanical hyperalgesia in rats, we did find clearcut hyperalgesia in rats with nerve ligation made with silk thread, thereby confirming our previous data^34^. Indeed, whereas Maves *et al*.^50^ placed ligatures sliding along the nerve, with no constriction, the ligatures’ tightness that is achieved following the original constriction procedure of Bennett and Xie^29^ has been regularly reported to produce strong mechanical hyperalgesia not only with chromic gut but also with silk thread around the nerve (see refs 51, 52, 53, 54). Accordingly, marked supersensitivity to mechanical stimulation of ipsilateral hindpaw was also observed after silk thread ligation of the sciatic nerve in CCI-SN rats used in the present study (Figs 3 and 4).

However, under acute treatment conditions, neither amitriptyline (12 mg/kg i.p.), mefloquine (1 mg/kg i.p.) nor their combination alleviated CCI-SN-induced mechanical supersensitivity. Because *in vitro*, apparent synergy was observed after rather long term exposure, for 24 h, of cultures astrocytes to these drugs, we then investigated the effects of a long term administration in CCI-SN rats.

Indeed, under chronic treatment conditions, a clear-cut interaction between the antidepressant and mefloquine was observed. As expected^55^,^56^,^57^, amitriptyline alone was found to reduce CCI-SN-induced hypersensitivity to mechanical stimulation and this effect was significantly enhanced in rats co-treated with mefloquine at a dose inactive on its own (2 × 0.5 mg/kg i.p. daily). The mefloquine-induced enhancement of amitriptyline effect was observed on both hindpaw withdrawal and vocalization responses, suggesting that underlying mechanisms involved both spinal and supraspinal relays of pain signaling^58^. The improved anti-hyperalgesic-like effect of amitriptyline could be linked to the reinforcement of its GJC inhibitory effect by mefloquine (Fig. 1), which might probably lead to an overall *in vivo* reduction of the release of pro-inflammatory compounds (such as ATP, glutamate, CXCL1) by astrocytes^9^,^10^,^11^,^12^,^13^,^14^. Indeed, at the doses administered in our studies, amitriptyline concentrations in brain reached the same μM range as those used in our *in vitro* assays with cultured astrocytes, supporting the idea that the apparent synergy observed *in vitro* between the tricyclic and mefloquine on Cx43-mediated GJC function might also occur *in vivo*. Interestingly, in both serum and brain, similar concentrations of amitriptyline were found whether or not rats had been co-treated with mefloquine, which allows us to rule out that the mefloquine effect would have resulted from some inhibition of amitriptyline catabolism.

Numerous studies have pointed out that antidepressants, including amitriptyline, exert anti-inflammatory properties^59^,^60^, which might contribute to their efficacy in alleviating pain^61^. Indeed, after neural lesion, microglia switches from a resting state to an activated state, with the production and release of pro-inflammatory cytokines, such as TNF-α, IL-1β and IL-6, which activate astrocytes^44^. Direct blockade of microglia activation by minocycline markedly attenuated nerve lesion-induced neuropathic pain^62^ and the decreased microglia production of proinflammatory cytokines by amitriptyline^59^, might as well underlay, at least partly, its anti-hyperalgesic action in CCI-SN rats. As HCs open in astrocytes exposed to pro-inflammatory cytokines released from microglia^13^,^14^, under our mixed astrocyte-microglia culture conditions, partial prevention of HC opening by amitriptyline might have resulted from the drug capacity to decrease microglia production of such cytokines.

These data led us to investigate whether the enhancement by mefloquine of the anti-hyperalgesic-like effect of chronic amitriptyline could be mediated through some increase of its anti-neuroinflammatory action. To this goal, we measured the concentrations of transcripts encoding relevant markers in both ipsilateral DRG and spinal cord tissues. Although these measurements were made four weeks after sciatic nerve ligation, mRNAs coding for ATF-3, a marker of neuronal injury^44^, and the proinflammatory cytokine IL-6, were still up-regulated in DRG. A tendency to some increase was also noted for the transcripts encoding GFAP, OX-42, IL-1β, but statistical significance was scarcely reached probably because of the marked decrease in corresponding genes induction four weeks following nerve ligation^44^. The progressive attenuation of neural lesion-induced neuroinflammatory reaction also very probably accounted for our finding that only OX-42 encoding transcript was still up regulated in spinal cord tissues four weeks after ligation. Interestingly, neither amitriptyline or mefloquine, nor their combination, were found to significantly affect the transcript levels of any of the neuroinflammatory markers in both DRG and spinal cord tissues. Although it cannot be excluded that some inhibition of CCI-SN-induced neuroinflammatory reaction might have occurred earlier, it seems rather unlikely that such an effect might have underlain the marked anti-hyperalgesic action of amitriptyline + mefloquine at the end of treatment. Possible implication of some change in Cx43 expression might also be ruled out because levels of its encoding transcript did not differ from respective values in control healthy rats. However, as emphasized above for other astrocyte markers^44^, this negative finding probably reflected the progressive attenuation of Cx43 induction after nerve lesion.

Altogether, these data suggest that mechanisms underlying the promoting action of mefloquine on the anti-hyperalgesic-like effect of chronic treatment with amitriptyline might target pain-signaling processes downstream of neural lesion-induced neuroinflammatory reaction. Further investigations at synaptic relays of nociceptive pathways in the dorsal horn of the spinal cord and supraspinal structures should allow deciphering how Cx modulation combined with a tricyclic antidepressant opens promising perspective toward improved treatment of neuropathic pain.

## Methods

### Animals

Male rats (weighing 175–200 g on arrival) of the Sprague-Dawley strain (Charles River breeding center, 69210 L’Arbresle, France), for which our laboratory has long expertise regarding neuropathic-like pain studies^34^,^35^,^44^, were accustomed to the housing facilities for one week before handling. Rats were housed under standard laboratory conditions (22 °C ± 1 °C, 60% relative humidity, 12-h light-dark cycle with light on at 7:00 a.m.) with free access to food and water *ad libitum*. On the other hand, the Wistar rat strain (Charles River) was selected for the preparation of cortical cell cultures (see below) from pups (P2), following the detailed procedure of Morioka *et al*.^28^.

All experiments were performed in strict conformity with the Ethical Guidelines of the Committee for Research and Ethical Issues of the International Association for the Study of Pain (IASP)^63^, the European Union laws and policies for use of animals in neuroscience research (European Committee Council Directive 86/609/EEC) and strictly followed the recommendations of the Ethical Committee of the French Ministry of Research and High Education (articles R.214-124, R.214-125; Registration nb.01296.01; official authorizations no. 006228 to S.B. and no. B75-116 to M.H., 31 December 2012). All experiments herein reported were specifically approved by the Ethical Committee for Preclinical Research (nb 5) of the French Ministry of National University Education and Research under the official notification nb R.214-987-126 of July 1, 2014. Throughout performance of the studies, all efforts were made to minimize the number of animals used and their suffering.

### Cell cultures

Brains were removed from Wistar rat pups, and cortices were dissected out. Meninges were carefully peeled off and cortical tissue was mechanically dissociated in phosphate buffered- saline (PBS, pH 7.4) supplemented with 33 mM glucose. Cells were seeded on polyornithine-coated plastic dishes at 5 × 10^5^ cells/dish of 35 mm in diameter or 3 × 10^6^ cells/dish of 100 mm in diameter (Nunc, Roskilde, Denmark), in medium containing DMEM (Sigma-Aldrich, St-Louis MO, USA), supplemented with penicillin (5 μg/mL), streptomycin (5 μg/mL) (Invitrogen, Carlsbad, CA, USA) and 10% fetal calf serum (FCS, Hyclone, Logan, UT, USA). After 8–10 days, when cells grown into 35 mm-dishes had reached confluence, 1 μM of cytosine-arabinoside was added to the culture medium during 3 days to eliminate proliferating microglial cells. Medium was then changed twice a week.

At confluence, cells grown in 100 mm-dishes were harvested with trypsin-EDTA (Invitrogen) and re-plated (2 × 10^5^ cells per well), as secondary cultures, on glass coverslips (Gassalem, Limeil-Brévannes, France) placed inside a 24-round-well plate (1.9 cm^2^/well, NunClon, Thermoscientific, Villebon-sur-Yvette, France). Finally, they were grown to confluence (about 1 week), cytosine-arabinoside was added and the medium was changed twice a week.

Treatments (with amitriptyline at 5, 10 or 20 μM, and/or mefloquine at 0.5 μM) consisted of cell exposure to drugs for 24 hours before GJ and HC functional assays.

### Scrape loading/Lucifer yellow transfer assay of gap junction channel activity

Cultured cells that had been treated as indicated above were first incubated at 20–22 °C for 10 min in HEPES buffered-saline 1 (HBS1: NaCl 140 mM, KCl 5.5 mM, CaCl_2_ 1.8 mM, MgCl_2_ 1 mM, d-glucose 10 mM, HEPES 10 mM, pH 7.35), then washed in Ca^2+^-free HEPES buffered-saline for 1 min. Cells were subsequently exposed to Lucifer yellow (LY, 1 mg/ml) for 1 min 11, washed, and LY was allowed to diffuse through GJCs during 8 min. Six successive fluorescent images taken at the center of the scrape line were captured using an inverted epifluorescence microscope (Diaphot-Nikon, Tokyo, Japan) for this 8 min diffusion period. Area of fluorescence was quantified with Image J program (NIH software).

### Ethidium bromide uptake assay of hemichannel activity

HC activity was evaluated by quantifying ethidium bromide (EtBr) uptake in cultured cortical astrocytes^64^. Briefly, as HC activity is low in basal condition, cells were pretreated with lipopolysaccharide (LPS, 1 μg/mL) overnight to enhance HC activity, thereby allowing optimal conditions to assess subsequent modulations by drugs^14^. The next day, they were pre-incubated in HEPES buffered-saline 2 (HBS2: NaCl 150 mM, KCl 5.4 mM, CaCl_2_ 2 mM, MgCl_2_ 1 mM, d-glucose 10 mM, HEPES 5 mM, pH 7.4), then incubated for 10 min in the same medium supplemented with 5 μM EtBr at room temperature, before washing and fixation with paraformaldehyde (4%). Ten consecutive images of fixed cells were taken at 0.49 μm intervals using a confocal laser-scanning microscope (Leica SP5), then stacked and finally analyzed with Image J program.

### Western blot

After treatment for 24 h with amitriptyline, mefloquine or both drugs together, cultured cells were harvested after scraping and centrifugation, and then cell pellets were suspended in 20 μL of 5X Laemmli sample buffer, boiled and sonicated (Ultrasonic cell disrupter, Microson). Proteins in sonicated samples (20 μg per sample) were separated by electrophoresis on Bis-Tris 4–12% NuPAGE gels and electro-transferred to nitrocellulose sheets. Nonspecific protein binding was blocked by incubation in TBS-Tween-milk solution for 1 hour. Blots were incubated overnight with primary anti-mouse Cx43 antibody (1:500, Transduction Laboratories, Lexington, KY, USA) at 4 °C, then with goat anti-mouse antibody conjugated to horseradish peroxidase (1:2,500, Tebu-Bio, 78610 Le Perray-en-Yvelines, France). Immunoreactivity was detected by ECL using the SuperSignal kit (Pierce, Rockford, IL, USA) according to manufacturer’s instructions. Blots were then reprobed with mouse monoclonal anti-glyceraldehyde 3-phosphate dehydrogenase (GaPDH) antibody (Sigma-Aldrich, 1:10,000) to check the protein load. Chemoluminescence imaging was performed on a LAS4000 (Fujifilm, Stamford, CT, USA). Semiquantitative densitometric analysis was performed with ImageJ software after scanning the bands.

### Chronic constriction injury (CCI) to the sciatic nerve (SN)

Rats were anesthetized with sodium pentobarbital (50 mg/kg i.p.), and the right common sciatic nerve was exposed at mid-tight level by blunt dissection through the biceps femoris muscle. Proximal to the trifurcation, ≈10 mm of nerve was freed from adhering tissue and four silk (5.0) ligatures were tied loosely (with ≈1 mm spacing) around the nerve. To obtain the desired degree of constriction, the criterion formulated by Bennett and Xie^29^ was used: the ligatures reduced the diameter of the nerve by a just noticeable amount and retarded, but did not interrupt, the epineurial circulation^34^. In the sham-operated rats, the right SN was exposed using the same procedure but was not ligated. The skin and muscle were finally sewed using silk sutures (4-0). After recovery from anesthesia on a warming pad in a postoperative room, CCI-SN and sham-rats were returned to their home cages until subsequent experiments.

### Pharmacological treatments

#### Acute treatments

Two weeks after surgery, CCI-SN rats received two injections two min apart. First, amitriptyline (12 mg/kg; Sigma-Aldrich, St Louis, MO, USA) or its vehicle (0.9% NaCl) was injected i.p. in a volume of 0.3 mL per CCI-SN rat. Then, CCI-SN rats were injected with mefloquine (1 mg/kg i.p.; Sigma-Aldrich) or its vehicle (0.9% NaCl with 0.017% DMSO).

#### Chronic treatments

Osmotic mini-pumps (Alzet, Charles-River) delivering amitriptyline at 12 mg/kg/day or 0.9% NaCl for 14 days were subcutaneously implanted in the back of CCI-SN rats under isoflurane anesthesia on the 15^th^ day post surgery. In addition, mefloquine (0.5 mg/kg) or its vehicle (0.9% NaCl with 0.017% DMSO) was administered i.p. twice a day (9:00 a.m., 6:00 p.m.) also for a 14-day treatment starting on the 15^th^ day post-surgery.

### Paw pressure test

Mechanical nociceptive thresholds, expressed as grams (g), were measured using an Ugo Basile analgesy-meter (Bioseb, 13127 Vitrolles, France) as described^35^. All determinations were made by an experienced person blind to treatment groups.

Briefly, an increasing pressure was applied onto the nerve-injured (right) hindpaw of CCI-SN rats until paw withdrawal and then vocalization (i.e. a squeak when the paw was maintained under pressure) were obtained. All tests were conducted between 10:00 a.m. and 5:00 p.m. in a quiet room where rats acclimatized for two hours before test performance. Basal responses were established on the day before nerve injury. On day 15 post surgery, when hypersensitivity to mechanical stimulation had fully developed^34^,^35^, responses thresholds were measured again to establish post-injury baseline, and further pressure threshold determinations were made at various times after subsequent acute or chronic treatments with amitriptyline and/or mefloquine or their vehicles (see above). Only animals showing clear-cut mechanical hypersensitivity (approximately 75% of CCI-SN rats), with a reduction of nociceptive thresholds of at least 30% compared to their pre-injury baseline, were used for these studies.

### LC/MS/MS quantification of amitriptyline in serum and brain

Rats were decapitated on the last day of chronic treatments, blood was collected from trunk vessels, and the brain was rapidly removed from the skull and frozen in isopentane at −30 °C before storage at −80 °C. Immediately after collection, blood samples were allowed to clot for 1 hour at 4 °C, and then centrifuged at 2,500 g for 25 min at 4 °C. Serum was collected and stored at −20 °C.

After thawing at 4 °C, serum samples were homogenized in perchloric acid (30%, v/v) and brain samples in a mixture of methanol and 10% perchloric acid (1:5, v/v). Precipitated proteins were sedimented by centrifugation at 10,000 × g for 10 min, and supernatants were collected. After extraction with a mixture of methanol: 10% formic acid (1:5, v/v), 20 μL aliquots (maintained in the auto-sampler kept at 4 °C) were injected into the liquid chromatography (LC) system (transcend TLX1, Thermo Fisher Scientific, San Jose, CA, USA). Chromatographic separation was carried out using a reverse-phase Hypersil GOLD column (50 × 2.1 mm, 1.9 μm, Thermo Fisher Scientific, San Jose, CA, USA) with the mobile phase consisting of a linear gradient of solvent A (0.05% formic acid in water) and solvent B (0.05% formic acid in acetonitrile) at a flow rate of 500 μL/min for a 10.90 min run. On-line MS analyses were performed using a 4000QTrap triple quadripole linear trap mass spectrometer equipped with a turbo ionspray source operated in electrospray mode (ABSciex, Foster City, CA, USA). The MRM transitions of m/z 278.2; 233.1 for amitriptyline and m/z 264.1; 233.2 for amitriptyline D6 (internal standard added in serum and brain homogenates before extraction step) were simultaneously monitored, and their respective concentrations calculated from specific peak surfaces (weight quadratic fit). Lower limit of quantification for amitriptyline was 10 μg/L. All determinations were made in triplicate for each sample.

### Real time RT-qPCR determinations of transcripts encoding neuroinflammatory and astrocyte markers

Rats were killed by decapitation on the last treatment day (see above), and both DRG and the dorsal quadrant of the lumbar enlargement of the spinal cord at L4-L6 level on the right side (ipsilateral to CCI-SN) were rapidly dissected out at 0–4 °C^44^. Dissected tissues were then immediately frozen in liquid nitrogen to be stored at −80 °C. Total RNA was extracted using the NucleoSpin RNA II extraction kit (Macherey-Nagel, 67722 Hoerdt, France) and quantified with a NanoDrop. First-stranded cDNA synthesis (from 660 ng total RNA per 20 μL of reaction mixture) was carried out using High capacity cDNA reverse transcription kit (Applied Biosystems, 91973 Courtaboeuf, France). PCR amplification, in triplicate for each sample, was performed using ABI Prism 7300 (Applied Biosystems), TaqMan^®^ Universal PCR Master Mix No AmpErase^®^ UNG (Applied Biosystems) and Assays-on-Demand Gene Expression probes (Applied Biosystems) for target genes: Activating Transcription Factor 3 (ATF3, assay ID Rn00563784_m1), Integrin alpha M (OX-42, Rn00709342_m1), GFAP (Rn01460868_m1), Interleukin-1β (IL-1β, Rn00580432_m1), Interleukin-6 (IL-6, Rn00561420_m1), Cx43 (Rn01433957_m1). Semi-quantitative determinations were made with reference to the reporter gene encoding glyceraldehyde 3-phosphate dehydrogenase (GaPDH; Rn99999916_s1). The polymerase activation step at 95 °C for 15 min was followed by 40 cycles of 15 sec at 95 °C and 60 sec at 60 °C^44^. The validity of the results was checked by running appropriate negative controls (replacement of cDNA by water for PCR amplification; omission of reverse transcriptase for cDNA synthesis). Specific mRNA levels were calculated after normalizing from GaPDH mRNA in each sample. Data are presented as relative mRNA units compared with control values^44^.

### Statistical analyses

All values are expressed as means ± S.E.M. Areas under the time-course curves (AUC) were calculated using the trapezoidal rule. Student’s-t-test, one-way ANOVA followed by Newman-Keuls test and Kruskal-Wallis test followed by Dunn’s post test, were used for analyses, except in case of paw pressure tests for which two-way ANOVA for repeated measures (effect over time) followed by Bonferroni test were used. For qRT-PCR data, the 2^−ΔΔCt^ method^65^ was used (RQ Study Software 1.2 version; Applied Biosystems).

## Additional Information

**How to cite this article**: Jeanson, T. *et al*. Potentiation of Amitriptyline Anti-Hyperalgesic-Like Action By Astroglial Connexin 43 Inhibition in Neuropathic Rats. *Sci. Rep.* **6**, 38766; doi: 10.1038/srep38766 (2016).

**Publisher's note:** Springer Nature remains neutral with regard to jurisdictional claims in published maps and institutional affiliations.
